# Time of day is associated with federal highway accidents in Brazil

**DOI:** 10.1590/1414-431X2025e14840

**Published:** 2026-01-30

**Authors:** V.C. Parro, C.R.C. Moreno, S. Folkard, L.B. Cardoso, D.D.V. Gueter, P.G.J. Mesquita

**Affiliations:** 1 Núcleo de Sistemas Eletrônicos Embarcados, Instituto Mauá de Tecnologia, São Caetano do Sul, SP, Brasil; 2 Departamento de Saúde e Sociedade, Faculdade de Saúde Pública, Universidade de São Paulo, São Paulo, SP, Brasil; 3 School of Psychology, Faculty of Medicine, Health and Life Science, Swansea University, Sketty, Swansea, United Kingdom

**Keywords:** Accident risk, Time of day, Single-vehicle accidents, Circadian, Fatigue, Safety

## Abstract

According to the most recent Global Status Record of Road Traffic, the number of road traffic deaths continues to rise. The risk of a road traffic death is more than 3 times higher in low-income countries than in high-income countries. However, the effect of time of day on road accidents is barely considered in public policies to reduce the chance of an accident. This study aimed to estimate the chance of an accident for every hour of the day in Brazil. From the raw data on accidents, their hourly distribution was derived, with a one-hour resolution. The data on the flow of vehicles on the highways was similarly organized. In the specific case of the flow, the total average flow for all sensors on Brazilian highways from 2015 to 2017 was used. It was clearly observed that the chance of an accident, in general, is on average 3-3.5 times higher between 02:00 and 04:00 h than during 07:00-19:00 h. Two other peaks were also noticed, the first one at around 07:00 h and a second one around 18:00 h, which were linked to an excess of vehicle traffic (rush hours) but were lower when compared to the chance during the night. The chance of a road accident in the middle of the night was higher compared to the rest of the 24 hours, similar to high-income countries.

## Introduction

According to the 2018 global status report on road safety from the World Health Organization (WHO), the number of deaths in 2016 due to traffic accidents reached 1.35 million, with low-income countries having a higher risk of death than high income countries (1). Although the number of deaths has decreased, the Sustainable Development Goal 3.6, which aimed to reduce the number of road traffic deaths by 50% by 2020, has not been achieved. Brazil is a middle-income country where the number of recorded road accidents has changed since 2015 when the recording of accidents became mandatory only when there were victims (2). However, Brazil still ranks fifth among countries with the highest number of deaths from road traffic injuries (3). A recent study showed that non-capital cities in Brazil had unfavorable trends in standardized mortality rates due to road traffic injuries, with accidents involving motorcycles being the most common (4). While some regions in Brazil showed a reduction in mortality rates, others showed a steady increasing trend or an increase in mortality rates due to road traffic injuries.

Excessive daytime fatigue and sleepiness are commonly caused by sleep disorders, including obstructive sleep apnea syndrome (OSAS), periodic limb movement disorder (PLMD), narcolepsy, and insomnia (5,6). Sleep-related car crashes represent up to 20% of all traffic accidents in industrialized societies, and driving drowsy has been identified as the major contributor to fatal road crashes. Studies indicate that more drivers experience falling-asleep crashes between 06:00 and 17:00 h than during the night. However, these results do not consider the flow of vehicles, which is lower at night compared to daytime (7). Another study about the time of day distribution of single-vehicle nodding-off crashes, excluding incidents involving alcohol and controlled substances, found that younger drivers experienced peak crash occurrence at night, while older drivers experienced it during the day (8). The 24-h pattern in drowsy-driving crashes has been explored during the past decades (9,10). On the other hand, a recent study demonstrated that time of day variations indicate more severe injuries at night, particularly when drivers are under the influence of alcohol or drugs (11).

Considering the lack of studies investigating the association between time of day and road accidents in Brazil, and the relevance of this factor to reduce road traffic injuries, this study aimed to estimate the chance of an accident in Brazil, correcting the raw data by the flow of vehicles by time of day.

## Material and Methods

This section describes the general scenario of road accidents in Brazil from 2015 to 2017 using consolidated data. One of the reasons for choosing this period was that, from 2015 onward, accident recording became more flexible, and registration became mandatory only when there were victims; even so, the number remains significant and serves as an important reference. To illustrate the effect of this measure, 186,745 and 169,197 accidents were recorded in 2013 and 2014, respectively, and 122,158 and 96,362 in 2015 and 2016, respectively. In addition to this policy change, the Brazilian economic crisis certainly had an impact on vehicle circulation. Table 1 summarizes the main indicators for the 2015-2017 period, from the Federal Highway Police (PRF) database. Despite the change in absolute numbers, the proportions between the years are quite steady.

**Table 1:** Accidents in Brazil in 2015, 2016, and 2017.

Year	Victims				Time of day				Road feature		
	Total	Fatal	Wounded	No victims	Early morning	Early night	Night	Day	Curve	Straight road	Crossroad
2015	122,158	4.62%	46.31%	47.81%	5.37%	5.73%	31.23%	57.66%	21.64%	72.49%	5.86%
2016	96,362	5.56%	56.94%	35.78%	5.66%	5.62%	33.49%	55.23%	23.13%	71.58%	5.30%
2017	89,507	5.79%	59.89%	34.32%	5.23%	5.44%	33.73%	55.60%	19.08%	60.16%	4.26%

**Figure 1:**
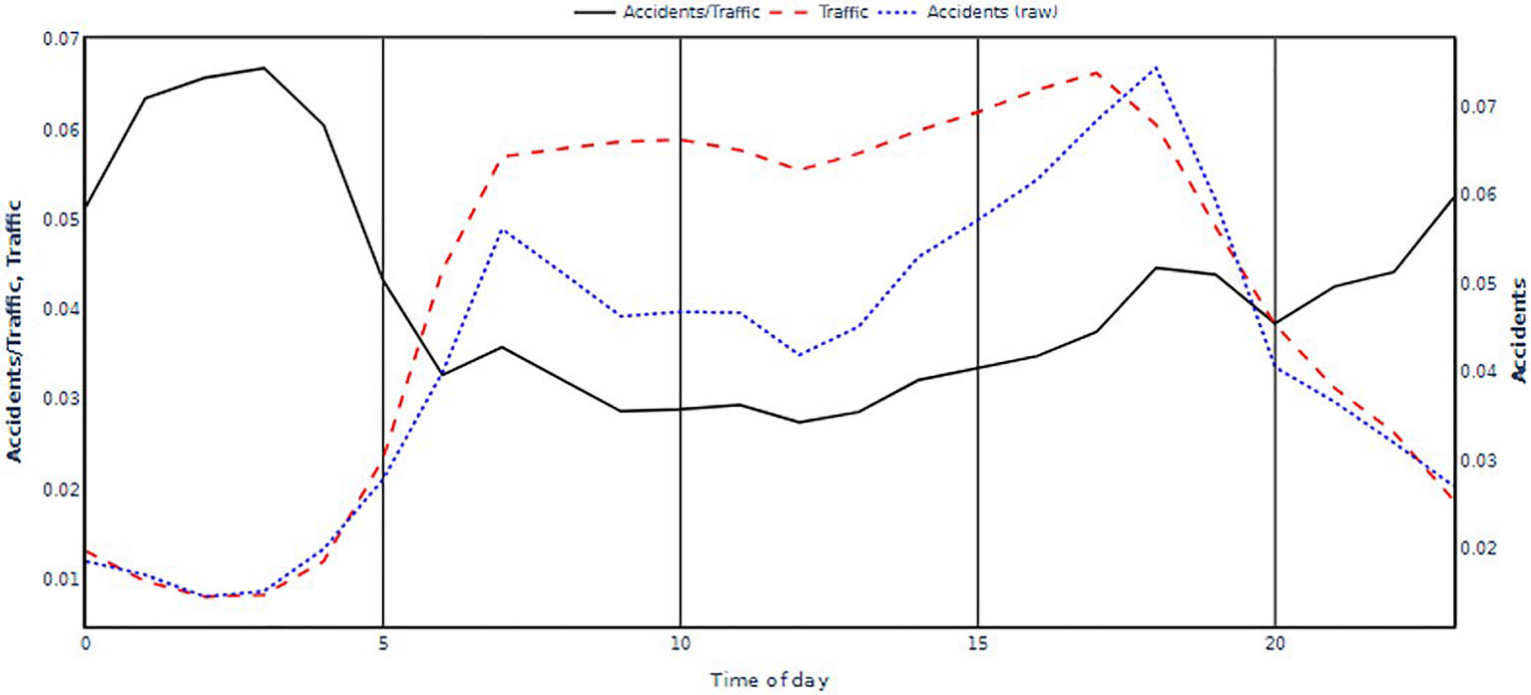
Hourly distribution of accidents and traffic flow where the solid line refers to $\frac{1}{3}\sum_{k=1}^{3}\frac{acc_{k}(h)}{flw_{k}(h)}$ ; dashed line: $\frac{1}{3}\sum_{k=1}^{3}acc_{k}(h)$ ; and dotted line: $\frac{1}{3}\sum_{k=1}^{3}flw_{k}(h)$

The hourly distribution of accidents was derived from the raw data provided by the PRF with a one-hour resolution. To facilitate notation, the index k was used to indicate the year, where: k=1 refers to 2015, k=2, 2016, and k=3 2017. In this sense, 3 discrete curves for accidents were obtained, with 24 points each, indicated by acck(h), where h is the hour of the day. Figure 1 shows the average behavior of accidents over the three years during the day.

Highway traffic flow was estimated based on data from the National Department of Transport Infrastructure (DNIT). These data are collected by sensors positioned on the highways and are grouped by hour in the same way as accidents. In this sense, 3 discrete curves for traffic flow were obtained, with 24 points each, indicated by flwk(h). Figure 1 shows the average behavior of traffic flow over the three years of flow throughout the day.

It can be said that the flow of one year consistently explains the flow of the other years. These aspects indicate stability of the flow and its variability in the years studied. The R-squared metric was used to justify the practical equivalence of two datasets over different years. Specifically, the R^2^ij value was calculated, where i denotes the dataset from years 1, 2, or 3, and j denotes the reference year for comparison. This determines how well the dataset from year i is explained by the dataset from year j. By comparing the flow between the base years used in this paper, the results shown in Table 2 were determined.

**Table 2:** Comparison of traffic flow between the years of this study - R^2^_ij_.

Years	R^2^_ij_ - Comparing flw_k_(h)	P-value
2016-2015	R^2^_21_=0.99979	6.8·10^-42^
2017-2016	R^2^_31_=0.99983	7.9·10^-43^
2018-2017	R^2^_32_=0.99954	3.0·10^-38^

Statistical test: R-squared.

## Results

To estimate the chance of an accident [c(h)], the raw data [acck(h)] was corrected by traffic flow [flwk(h)], using the equations shown in the legend of Figure 1, and c(h) is shown in the overlapping graphs of Figure 1. This operation shows the relationship between accidents and time of day. As expected, the absolute number of accidents is higher when there is a higher flow of vehicles, so that the ratio of accidents per time of day must be corrected for traffic flow.

The results showed that the chance of an accident, in general, was higher in the middle of the night, with the peak being between 01:00 and 03:00 h. Two other peaks were also found, the first one at around 07:00 h and a second one around 18:00 h, which were linked to an excess of vehicle traffic (rush hours), but were lower when compared to the risk at night. Another important aspect was that the accident data were consistent when the years were compared individually, and it can be said that the ck(h) was stable over the years (Table 3).

**Table 3:** Comparison of accidents between the years of this study - R^2^_ij_.

Years	R^2^_ij_ - Comparing c_k_(h)	P-value
2016-2015	R^2^_21_=0.95909	9.2·10^-17^
2017-2016	R^2^_31_=0.90242	1.3·10^-12^
2018-2017	R^2^_32_=0.92035	1.4·10^-13^

Statistical test: R-squared.

Figure 1 shows an increase in the risk of accidents from 02:00 h onwards, reaching its maximum at 04:00 h. On average, the risk of accidents at these early hours was about 3-3.5 times greater than during normal working hours. However, when the results from individual years were examined, it was observed that, for example, in 2015 there were two distinct peaks, the first at 02:00 h and the second at 04:00 h. The first peak is in accordance with a previous study (12) and may be due to alcohol consumption while the second may reflect human error, sleepiness, and fatigue.

**Figure 2:**
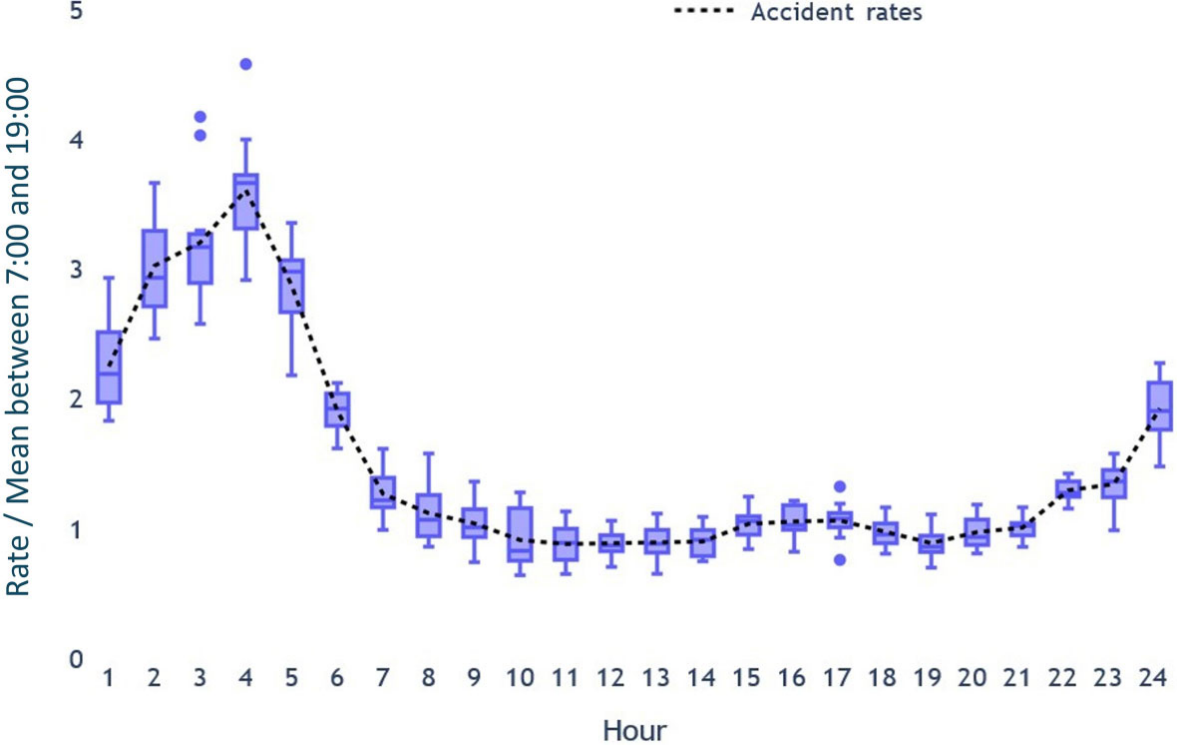
Normalized accident rates per hour, achieved by dividing the rate by the mean number of accidents between 07:00 and 19:00 h. Data are reported as median and interquartile range.

In order to estimate the contribution of sleepiness and fatigue to the early-morning peak in accidents shown in Figure 1, four non-overlapping types of accidents were examined: single vehicle rollovers or tippings on straight or curved roads. Combined across the three study years, these four types of single-vehicle accidents yielded 12 values for each hour of the day, allowing the estimation of confidence intervals. To assess how much greater the chance of accidents was during the early morning hours compared to typical daytime driving, the values were normalized by dividing them by the average number of accidents between 07:00 and 19:00 h. Figure 2 shows a significant increase in accident risk from 02:00 h onwards, peaking at 04:00 h. On average, the risk of accidents in the early morning hours was about 3-3.5 times greater than during normal working hours.

## Discussion

The findings of this study clearly indicated a higher chance of vehicle accidents between 01:00 and 03:00 h. These results are consistent with findings from a recent study with 35 bus drivers, which found the highest subjective sleepiness score at 04:00 h (±1 h) (13). In addition, these authors found an effect of time of day on working memories, with the lowest working memory scores at 04:00 h (±1), being sleepiness significantly associated with decreased working memory. A systematic review with ten cross-sectional studies (51,520 participants), six case-control studies (4904 participants), and one cohort study (13,674 participants) has recently shown driver sleepiness was associated with an increased risk of motor vehicle accidents (14).

Professional drivers who work at night and are prone to sleep disorders such as obstructive sleep apnea (OSA) (15,16) have reported sleepiness while driving. However, noncommercial drivers, regardless of whether they have OSA, also report sleepiness at the wheel (17,18). In Brazil, the prevalence of OSA in railroad workers was found to be higher than that observed in the general population (19). Sleepiness, reduced alertness, and sleep disorders probably explain the increased risk of nighttime accidents found in this study.

In addition to the factors previously reported in the literature, the pronounced nighttime peak in accident probability - particularly between 01:00 and 03:00 h - may result from a combination of physiological and contextual factors specific to the Brazilian setting. This period coincides, in general, with the circadian nadir, when homeostatic and circadian drives for sleep reduce alertness and cognitive performance. In Brazil, a significant proportion of nighttime highway traffic consists of professional drivers, such as truck and bus operators, who are frequently subjected to extended working hours and irregular sleep-wake patterns due to demanding schedules.

These conditions are associated with increased sleep pressure and a higher prevalence of fatigue-related impairments. Additionally, the presence of undiagnosed or poorly managed sleep disorders such as sleep apnea in this occupational group may further increase the risk of nighttime accidents (17). Importantly, although alcohol consumption is a well-known risk factor for traffic accidents, its contribution in this context is likely minimal due to Brazil's strict zero-tolerance policy for alcohol in traffic enforcement and the widespread use of breathalyzer checks by transportation companies prior to departure. Therefore, fatigue and circadian misalignment appear to be the most plausible contributors to the elevated risk of accidents observed during nighttime.

The present study also found two peaks of accidents during the day: the first one at around 07:00 h and a second one around 18:00 h. These peaks are linked to excess traffic (rush hours) and were lower than the number of accidents at night.

To date, no study in Brazil has estimated the likelihood of a road accident as a function of the time of day. The results also suggest that a rollover accident involving a single vehicle on a straight road may suggest human error as the cause of the incident. In such cases, it may not be necessary to rely on the police report to reach this conclusion. By isolating and analyzing these types of accidents, the underlying factors can be investigated, and targeted preventive measures can be taken to reduce their occurrence in the future.

The findings of this work may have important implications for road safety policy, particularly regarding nighttime highway risks. One actionable measure would be the expansion and strategic distribution of safe and well-equipped rest areas along major highways. In several regions of Brazil, the distance between rest stops is considerable, limiting drivers' ability to take restorative breaks. Increasing the number and accessibility of these facilities would support compliance with rest regulations and reduce fatigue-related accidents. In addition, increasing public awareness of risks associated with fatigue by means of warning notices on highways that encourage drivers to use the rest stops (such as the UK's “Tiredness kills - take a break“) may reduce fatigue-related accidents.

Another critical aspect involves addressing the use of stimulant substances, which are sometimes clandestinely sold at gas stations and roadside establishments. These substances are often used by professional drivers as a coping mechanism to stay awake during long trips, but they can impair judgment and increase accident risk. Strengthening surveillance and enforcement against the illegal sale of stimulants in such locations could play a key role in reducing nighttime crash rates.

Finally, improving the reports of accidents caused by driver sleep episodes is essential. Underreporting or misclassification of these incidents limits our ability to generate reliable statistics and identify high-risk factors. Establishing clearer protocols for crash investigation and attributing fatigue as a causal factor would allow for more targeted interventions and better resource allocation. Together, these policy actions - enhancing rest infrastructure, enforcing stimulant regulations, and improving data quality - could significantly mitigate nighttime accident risks and inform more effective law enforcement scheduling and driver education campaigns.

## Conclusions

This research supports the findings of previous studies conducted in other countries, indicating that Brazilian highways and vehicles require measures to prevent accidents at night. In addition, this study highlights the importance of implementing control measures to improve road safety and reduce the risk of accidents during nighttime driving, suggesting some possible policy implications based on the results.

## Data Availability

The data used in this paper can be found on the following website links: Change of mandatory accidents recording in Brazil <https//www.gov.br/transportes/pt-br/assuntos/dados-de-transportes/bit/publicacoes-1/Anuario%20Estatistico%20de%20Seguranca%20Rodoviaria.pdf/view>; Traffic accidents data: <https//www.gov.br/prf/pt-br/acesso-a-informacao/dados-abertos-da-prf>; Vehicles flow: https://www.gov.br/dnit/pt-br; Python codes: <https://github.com/vparro/Mlprobabilistico/blob/master/Paper_accidenets_data.ipynb>.
